# Genome sequence and effectorome of *Moniliophthora perniciosa* and *Moniliophthora roreri* subpopulations

**DOI:** 10.1186/s12864-018-4875-7

**Published:** 2018-07-03

**Authors:** Ceslaine Santos Barbosa, Rute R. da Fonseca, Thiago Mafra Batista, Mariana Araújo Barreto, Caio Suzart Argolo, Mariana Rocha de Carvalho, Daniel Oliveira Jordão do Amaral, Edson Mário de Andrade Silva, Enrique Arévalo-Gardini, Karina Solis Hidalgo, Glória Regina Franco, Carlos Priminho Pirovani, Fabienne Micheli, Karina Peres Gramacho

**Affiliations:** 1 Departamento de Ciências Biológicas (DCB), Centro de Biotecnologia e Genética (CBG), Universidade Estadual de Santa Cruz (UESC), Rodovia Ilhéus-Itabuna, km 16, Ilhéus, 45662-900 Bahia Brazil; 2 Comissão Executiva do Plano da Lavoura Cacaueira (CEPLAC), Centro de Pesquisas do Cacau (CEPEC), Seção de Fitossanidade (SEFIT), Laboratório de Fitopatologia Molecular (FITOMOL), km 22 Rod. Ilhéus Itabuna, Ilhéus, 45600-970 Bahia Brazil; 3 The Bioinformatics Centre, Department of Biology, University of Copenhagen, Copenhagen, Denmark; 4 CIMAR/CIIMAR, Centro Interdisciplinar de Investigação Marinha e Ambiental, Universidade do Porto, Porto, Portugal; 5 Departamento de Bioquímica e Imunologia, Universidade Federal de Minas Gerais/Belo Horizonte, Minas Gerais, Brazil; 6 Instituto de Cultivos Tropicales –ICT, Tarapoto, Peru; 7 Instituto Nacional de Investigaciones Agropecuarias – INIAP, Departamento de Protección Vegetal, Quito, Ecuador; 8 CIRAD, UMR AGAP, F-34398 Montpellier, France

**Keywords:** *Theobroma cacao*, Witches’ broom, Frosty pod rot, Pathogenicity factors, Plant pathogens

## Abstract

**Background:**

The hemibiotrophic pathogens *Moniliophthora perniciosa* (witches’ broom disease) and *Moniliophthora roreri* (frosty pod rot disease) are among the most important pathogens of cacao. *Moniliophthora perniciosa* has a broad host range and infects a variety of meristematic tissues in cacao plants, whereas *M. roreri* infects only pods of *Theobroma* and *Herrania* genera. Comparative pathogenomics of these fungi is essential to understand *Moniliophthora* infection strategies, therefore the detection and in silico functional characterization of effector candidates are important steps to gain insight on their pathogenicity.

**Results:**

Candidate secreted effector proteins repertoire were predicted using the genomes of five representative isolates of *M. perniciosa* subpopulations (three from cacao and two from solanaceous hosts), and one representative isolate of *M. roreri* from Peru. Many putative effectors candidates were identified in *M. perniciosa*: 157 and 134 in cacao isolates from Bahia, Brazil; 109 in cacao isolate from Ecuador, 92 and 80 in wild solanaceous isolates from Minas Gerais (Lobeira) and Bahia (Caiçara), Brazil; respectively. *Moniliophthora roreri* showed the highest number of effector candidates, a total of 243. A set of eight core effectors were shared among all *Moniliophthora* isolates, while others were shared either between the wild solanaceous isolates or among cacao isolates. Mostly, candidate effectors of *M. perniciosa* were shared among the isolates, whereas in *M. roreri* nearly 50% were exclusive to the specie. In addition, a large number of cell wall-degrading enzymes characteristic of hemibiotrophic fungi were found. From these, we highlighted the proteins involved in cell wall modification, an enzymatic arsenal that allows the plant pathogens to inhabit environments with oxidative stress, which promotes degradation of plant compounds and facilitates infection.

**Conclusions:**

The present work reports six genomes and provides a database of the putative effectorome of *Moniliophthora*, a first step towards the understanding of the functional basis of fungal pathogenicity.

**Electronic supplementary material:**

The online version of this article (10.1186/s12864-018-4875-7) contains supplementary material, which is available to authorized users.

## Background

Witches’ Broom (WB) and Frosty Pod Rot (FPR) diseases of cacao; respectively caused by *Moniliopththora perniciosa* (Stahel) Aime Phillips-Mora (2005) and *Moniliophthora roreri* H. C. Evans, Stalpers, Samson & Benny [1], are among the most devastating diseases affecting cacao plantations. Yield losses are usually over 30%, but can reach 100% in some circumstances, leading to the total abandonment of cacao cultivation. WB caused a near collapse of cacao farming in Bahia state, Brazil. FPR is a quarantine disease in Brazil, and although it is still not reported in the country, there is a great risk of its spread into the cacao-producing areas of Brazil due to their proximity with countries in which the disease is present.

Both *M. perniciosa* and *M. roreri* (Phylum Basidiomycota; Order Agaricales; Class Agaricomycetes; Family Marasmiaceae) [1] are hemibiotrophic fungi with similar lifestyle and two distinct colonization phases. The biotrophic phase characterized by convoluted mycelium that colonizes the intercellular space, and the necrotrophic phase characterized by hyphae that invades the cells leading to internal and external necrosis and death of the infected tissues [2, 3], and fungal sporulation. Although these pathogens share some commonalties, there are differences that discriminate them.

*Moniliophthora perniciosa* is able to infect a variety of meristematic tissues: vegetative shoots, flower cushions, flowers and cacao pods. The most characteristic symptom of an infection with *M. perniciosa* is the hypertrophic growth of the infected vegetative meristem, shaped like a broom (hence the name) [4]. The infective propagule of *M. perniciosa* is a basidiospore produced in the lamellae of the basidiomata that emerge from the dead plant tissue [2]. On the other hand, M. roreri is pod specific [5], and the spores are produced on thick, felt-like pseudostroma, which are powdery when mature without the formation of basidiomata. The amount of spores produced by *M. roreri* combined with their longevity have largely contributed to its ability to invade new territories [6].

*Moniliophthora roreri* is only able to infect individuals of *Theobroma* and *Herrania*, two genera of the Malvaceae family. *Moniliophthora perniciosa* has a much wider range of plant hosts encompassing both plant species from the Malvaceae family and distantly related families, e.g. the Solanaceae family. Four biotypes, based on the pathogen ability to infect a particular plant species have been recognized [7]: biotype C is specific to the Malvaceae family, infecting the genera *Theobroma* and *Herrania*; biotypes S and L infect Solanaceae and many species of vines and lianas from the Malpighiaceae and Bignoniaceae families; and the biotype B which exclusively infects *Bixa orellana* (Bixaceae). Previous studies analyzed the karyotype of *M. perniciosa* and assessed their diversity using molecular and biochemical markers, uncovering genetic similarity between biotypes C and S. High variation at chromosomal level and in microsatellite telomeric profiles among isolates of biotype C were observed [8]. Global population genetics analyses, using 11 microsatellite markers well-characterized *M. perniciosa* isolates from biotypes C and S, reported the existence of five host genetically distinct *M. perniciosa* subpopulations in Brazil [9]. These genetically differentiated host subpopulations have unique host associations and a high degree of both host and cultivar specificity [9, 10]. Isolates originating from cacao always cause infection in cacao plants, but not necessarily on a solanaceous host, whereas some solanaceous isolates, e.g., from Lobeira, proved to be nonpathogenic in cacao [9].

Fungal plant pathogens interact largely with their plant hosts via the secretion of effectors. Fungal effectors are small molecules associated with an organism that manipulate host cell physiolocal and morphological processes in the plant hosts. Thus facilitating infection (virulence factors or toxins) and/or provoking plant defenses (avirulence factors: Avr) [11]. Most of the identified eukaryotic pathogenic effectors do not contain domains or homologies to proteins with known function; therefore, their roles remain unclear. In general, fungal effectors are highly polymorphic, a characteristic attributed to their rapid adaptation to the host [12]. Most of these are rich in cysteine, from multigenic families and from specific lineage [13]. The in silico identification and functional characterization of these proteins will be the first step towards identifying the mechanisms of colonization by the different host subpopulations adding knowledge about the biology and modes of action of these host specific subpopulations.

Fungal genomes of isolates with specific adaptations (e.g., as a function of habitat and host) are expected to be molded according to the infection strategies employed by the pathogen in order to maximize the success for pathogenicity, i.e., its ability to provoke the infection. Therefore, the availability of genomic data from different isolates of the same pathogen is essential to uncover genomic variation intrinsic to the pathogenicity of certain species, subpopulation or fungal populations [14].

Whole-genome sequencing, determined by bioinformatics/statistical methods, has become a method of choice to perform genome-scans for candidate effectors across isolates and/or species, particularly in obligate biotrophs where functional approaches are impeded. The currently available *M. perniciosa* genome (isolate 553) [15], generated by a consortium of Brazilian Institutions (www.lge.ibi.unicamp.br/vassoura), revealed that the pathogen contains a 26.66-Mb genome organized in 8 chromosomes with 13,560 predicted proteins [16]. The analysis allowed a general overview of the *M. perniciosa* genome highlighting important genes involved in stress adaptation, plant necrosis induction and genes associated with pathogenesis mechanisms [15]. Rincones et al. [17] carried out a comparative transcription analysis between biotrophic and saprophytic *M. perniciosa* phases found specific genes at each stage of its life cycle. For example, oxaloacetate acetyl hydrolase in the biotrophic phase, putative virulence genes (e.g., glucuronyl hydrolase; putative chitinase) and transposons (induced in the biotrophic phase) [2, 18]. A full genome of *M. roreri* from an isolate collected in Ecuador revealed a genome with 52.3 Mb and 17,910 predicted genes [4] that showed 93% similarity with genes encoding secreted proteins in *M. perniciosa*. Sequencing of more distinct isolates from *M. roreri* and *M perniciosa* subpopulations will help to gain more information on the biology of these pathogens, contributing to the prevention of FPR in Brazil as well as to better understanding WB caused by isolates other than cacao.

In this context, comparative pathogenomics can be an important tool for understanding *Moniliophthora* infection strategies. With the availability of the reference genomes for *M. perniciosa* and *M. roreri*, we report genomes of six *Moniliophthora* isolates: i) two isolates of *M. perniciosa* that differ in pathogenicity level to cacao plants; ii) one *M. perniciosa* isolate from Ecuador; iii) two *M. perniciosa* isolates representative of the host subpopulations previously defined by Patrocínio et al. [9]; and iv) one *M. roreri* isolate representative from Peru (Bolivar group according to Phillips-Mora et al. [19]). The power and usefulness of these genome scans provides an important step to prioritize candidate effectors of interest for future studies.

## Methods

### *Moniliophthora perniciosa and M. roreri* isolates and DNA isolation

In the present work we used five *M. perniciosa* genomes; representative of previously described subpopulations within the Solanaceae (2) and of the Malvaceae (3) families [9, 20–22], and one *M. roreri* genome obtained from *Theobroma cacao* at the Instituto de Cultivos Tropicales (ICT), Peru.

Each isolate is specific of subpopulation: **Mp4145** (CEPLAC/CEPEC, Bahia, Brazil accession number 4145) and **Mp1441** (CEPLAC/CEPEC, Bahia, accession number 1441) isolated from a susceptible cacao genotype collected in 2003 and 2012, respectively, and represents two separate incursions of *M. perniciosa* in Bahia [20]. **Mp178** (CEPLAC/CEPEC, Bahia, accession number 4413) and **Mp4071** (CEPLAC/CEPEC, Bahia, accession number 4071) were derived from the wild solanaceous hosts lobeira in Minas Gerais and Caiçara in Bahia (both from Brazil); that do not infect cacao [21]. **Mp4124** (INIAP/Ecuador, accession number 404) is a representative isolate from *M. perniciosa* population’s from Ecuador [22], and **MrPeru** (Peru/ICT, accession number 05) is a representative of one of the major groups of *M. roreri* (the Bolívar group) established by Phillips-Mora et al. [19] in a global diversity study. For simplicity, hereafter these are referred to as “isolates”.

Isolates from Bahia have been maintained as viable cultures in the *M. perniciosa* (CEPLAC/CEPEC/FITOMOL) culture collection (CEGEN N° 109/2013/SECEXCGEN) in sterile distilled water [23] and in mineral oil. Foreign isolates from Ecuador and Peru were received as pure DNA.

The genomic DNAs were extracted from 2 g of mycelial fresh mass using the AxyPrep Multisource Genomic DNA Kit (AxyGen, CAT. N° AP-MN-MS-GDNA-50, Union City, CA, USA). DNA of *M. roreri* isolate was obtained from ICT, Peru. The concentration and quality of the DNA obtained were checked in Qubit and NanoDrop™ 8000 Spectrophotometer (Therm Fisher Scientific) in 1% agarose gel. The identities of the isolates were validated using the highly conserved fungal rRNA gene primers (ITS1F and ITS4) as previously described [21, 24].

### Data filtering, de novo assembly and mapping sequencing

Genomes of *M. perniciosa* and *M. roreri* (MrPeru) isolates were sequenced at the Center of Biotechnology and Genetics (CBG), UESC/Laboratory of Molecular Markers, in Bahia, Brazil using Illumina MiSeq® platform. The DNA was used to generate Illumina shotgun paired-end sequencing libraries prepared with the Nextera DNA Sample Preparation/illumina® (CAT. N° FC-121-9009) following the manufacturer instructions and sequenced by Illumina MiSeq® reagents kit V3 600 cycles (Illumina®, CAT. N° 15,043,894). Libraries were validated and quantified with KAPA Library Quantification Kit Illumina® Platforms (KR0405 v6.14), in ABI Prism real-time PCR according to the manufacturer protocol. The PhiX, a standard of 10 Nm and 500 pb, was used to ensure absolute quantification of the libraries. The concentration and quality of the libraries were inferred by the dissociation curve analysis of the graph obtained after qPCR, wherein the presence of adapter dimers was also evaluated. The reads were filtered with the FastQC software. Repeat Masker v4.0.1 software [25] was used to identify repetitive elements. Quality and completeness of genome was evaluated using Benchmarking Universal Single-Copy Orthologs Version 2 (BUSCO v2) based on a Basidiomycota ortholog dataset [26]. Prediction of genes was performed with the Augustus software v3.2.3 [27]. An annotation pipeline, MAKER2 [28], was used to choose the best possible gene model based on evidence alignments. The Mp4145 sequence is available at the UESC-CEPLAC restricted databases at http://nbcgib.uesc.br/mperniciosa.

### Phylogenomics

The phylogeny of the isolates was reconstructed based on a concatenated alignment of 610 orthologs and multiple sequence alignments were performed with MUSCLE software v3.8.31 [29]. A maximum likelihood tree was obtained with RAxML v8.0.9 [30] using the GTRGAMMA model with 1000 bootstrap replicates. iTOL - Interactive Tree of Life v4 software [31] was used to display the best-rated ML tree The MrPeru isolate (*M. roreri)* was used as an outgroup.

### Identification of candidate secreted effector proteins

**Fig. 1:**
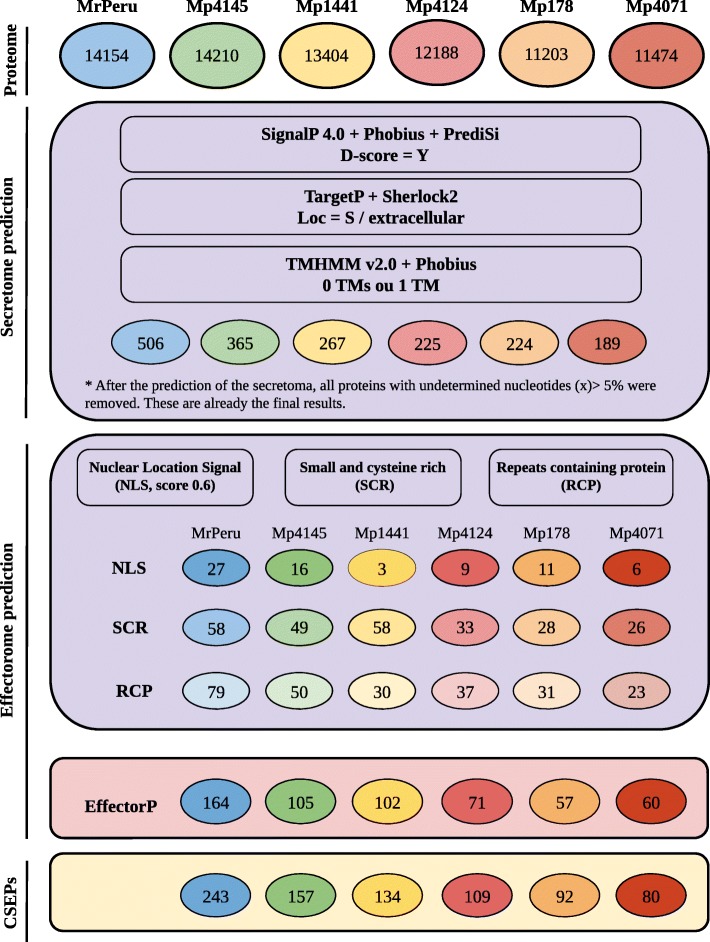
Pipeline for in silico characterization of candidate secreted effector proteins (CSEPs). The prediction of the secretoma was performed from the putative proteome. Effector candidates were identified from these secreted proteins that meet at least one of the following criteria: (I) Proteins with nuclear localization signal (NLS), (II) small (<= 150 aa) and cysteine (> 3%) (SCR) proteins, and (III) proteins containing repeats (PCR). They were also predicted by the EffectorP software. The set of CSEPs was formed with the sum of the proteins with NLS, SCR, and RCP and deduced by EffectorP (Pipeline adapted from Toro and Brachmann [38]). Proteins predicted more than once by the established criteria were counted only once. MrPeru - *Moniliophthora roreri* isolate from Peruvian subpopulation. Mp4145 and Mp1441 - *M. perniciosa* isolates from cacao subpopulations in Bahia. Mp4124 - *M. perniciosa* isolate from cacao subpopulation in Ecuador. Mp178 - *M. perniciosa* isolate from wild solanaceous subpopulation (Lobeira) in Minas Gerais. Mp4071 - *M. perniciosa* isolate from wild solanaceous subpopulation (Caiçara) in Bahia

Secreted proteins were characterized as proteins containing a signal peptide. Signal peptides were identified using three softwares: SignalP 4.1 [32], Phobius [33] and PrediSi [34], with D-score = Y. Protein subcellular localizations were conducted using TargetP [35] Loc = S and SherLoc2 [36] softwares with “extracellular” addressing parameter. TMHMM v2.0 [37] and Phobius [33] softwares were used to keep proteins with one Transmembrane domain (TM) or without TM located on the N-terminal signal peptide. To increase the stringency, only predicted proteins selected by both softwares were considered for further analyses. After the secretome prediction, proteins with 5% or higher of undetermined amino acids (X) were removed (Fig. 1).

Further, based on Toro and Brachmann [38] effectors prediction pipeline, secreted proteins were mined for candidate secreted effector proteins (CSEPs) considering at least one of the following effector-oriented criteria: (i) nuclear localization signal (NLS) proteins using NLStradamos [39], (ii) small proteins (<= 150 aa) rich in cysteine (> 3%) (SCR), using a perl script, and (iii) repeats containing protein (RCP), with the XTREAM software [40], To increase the likelihood of identifying effectors, CSEPs were also predicted by EffectorP software [41] (Additional file 1: Table S1; Fig. 1). Finally, we built a database of CSEPs. Proteins predicted by more than one criterion were counted only once.

Next, functional characterization of the CSEPs was carried out using BLAST2GO tool software [42]. The sequence similarity was obtained using the BLASTp algorithm against NCBI Non-Redundant Database (NR). The CSEPs annotation was performed using Gene Ontology (GO).

## Results

### De novo genome sequencing and phylogenomics

We selected two well characterized isolates from cacao, Mp4145 and MrPeru to build the M. perniciosa and M. roreri genome sequences. The assembly resulted in genome sizes of approximately 45 Mb: 47.01 Mb in Mp4145, 46.36 Mb in Mp1441, and 45.47 Mb in Mp4124; 45.17 Mb in MrPeru, 44.42 Mb in Mp4071, and 43.92 Mb in Mp178. The genome assembly comprised an average of 2158.66 contigs with N50 average of 0.084 Mb among isolates, and the longest scaffold size of 0.91 Mb for the genome of M. perniciosa and 0.53 Mb of M. roreri (Table 1). The genome qualities varied among isolates, MrPeru showed the highest completeness with 95.9%, and Mp1441 the lowest (66%) from a total of 1335 BUSCO groups searched (Additional file 2: Table S2). The most abundant repetitive elements in all isolates were long terminal repeats (LTRs). In total, the repetitive elements corresponded to percentages smaller than 1.4% in all genomes (Additional file 3: Table S3). The genome annotation using MAKER2 software [28] allowed us to predict 14,154 (MrPeru), 14,210 (Mp4145), 13,404 (Mp1441), 12,188 (Mp4124), 11,203 (Mp178) and 11,474 (Mp4071) proteins in each genome (Table 1).

**Table 1:** Genetic features of genomes

	MrPeru	Mp4145	Mp1441	Mp4124	Mp178	Mp4071
Assembled genome size (Mb)	45.17	47.01	46.34	45.47	43.92	44.42
N50 scaffold size (Kb)	56	87	90	90	92	92
NumN50	226	141	137	133	126	128
Longest scaffold size (Kb)	530	910	910	910	910	910
Number of contigs	2994	2676	2100	1967	1526	1689
GC %	47.8	47.7	47.7	47.8	48	47.9
Proteome predicted	14,154	14,210	13,404	12,188	11,203	11,474

Comparison of the assembly statistics of Illumina sequencing of the genome of *Moniliophthora* spp. MrPeru - *Moniliophthora roreri* isolate from Peruvian subpopulation. Mp4145 and Mp1441 - *M. perniciosa* isolates from cacao subpopulations in Bahia. Mp4124 - *M. perniciosa* isolate from cacao subpopulation in Ecuador. Mp178 - *M. perniciosa* isolate from wild solanaceous subpopulation (Lobeira) in Minas Gerais. Mp4071 - *M. perniciosa* isolate from wild solanaceous subpopulation (Caiçara) in Bahia

**Fig. 2:**
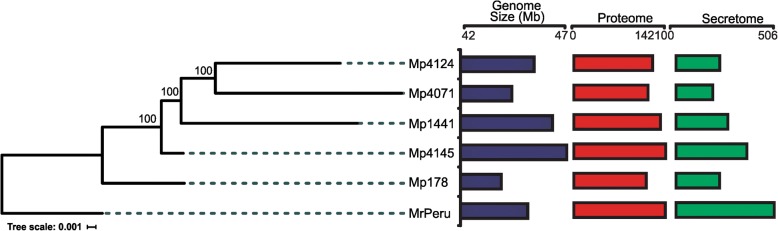
Phylogenomic tree of *Moniliophthora* isolates. The Maximum likelihood tree based on the alignment of concatenated nucleotides of 610 orthologs of unique copies of the genomes. Genome size (in blue), amount of putative proteome (in red) and putative secretome (in green) are shown for each isolate. Bootstrap values are 100% for all groupings. MrPeru - *Moniliophthora roreri* isolate from Peruvian subpopulation. Mp4145 and Mp1441 - *M. perniciosa* isolates from cacao subpopulations in Bahia. Mp4124 - *M. perniciosa* isolate from cacao subpopulation in Ecuador. Mp178 - *M. perniciosa* isolate from wild solanaceous subpopulation (Lobeira) in Minas Gerais. Mp4071 - *M. perniciosa* isolate from wild solanaceous subpopulation (Caiçara) in Bahia

The phylogeny was reconstructed based on a concatenated alignment of genes. The phylogenetic tree indicated a division of the isolates into two major clades: a clade containing *M. perniciosa* isolates from Ecuador (Mp4124) and Bahia (Mp4145 and Mp1441), as well as the wild solanaceous (Mp4071) isolate from Bahia; and another clade with the wild solanaceous isolate from Minas Gerais (Mp178), all supported with high bootstrap values (Fig. 2). Surprisingly, isolates Mp4124 and Mp4071, which came from different subpopulations and hosts, were rescued as a sibling group, being sibling clade of Mp1441. Mp4145 constitutes a clade with the grouping Mp1441, Mp4071 and Mp4145.

### Candidate secreted effector proteins

We combined multiple bioinformatic approaches to predict putative effectors within *M. perniciosa and M. roreri*, and those conserved across *Moniliophthora* species and isolates. Secreted proteins were accepted as candidate effectors if at least one of the following criteria was fulfilled: (i) nuclear localization signal (NLS), (ii) small proteins (≤150 aa) rich in cysteine (> 3%) (SCR), and (iii) repeats containing protein (RCP) [38, 43, 44]. In addition, we also used a software that searches for effector candidates using machine learning, the EffectorP [40]. This pipeline is outlined in Fig. 1. Concisely, the secretome of each isolate was predicted from the putative proteome using a series of combined softwares. Proteins with signal peptide in the N-terminal region addressing secretion and not being retained in the transmembrane region were predicted as secreted proteins. To achieve that, the results obtained individually from each program were combined, and the common sequences among the analyses for each category were selected as candidate secreted effector proteins (CSEPs). The predicted secretomes of the isolates were composed of 506 proteins from MrPeru, 365 from Mp4145, 267 from Mp1441, 225 from Mp4124, 224 from Mp178 and 189 from Mp4071.

Among the predicted effectors that contain NLS, 27 proteins were found in MrPeru, 16 in Mp4145, 3 in Mp1441, 9 in Mp4124, 11 in Mp178 and 6 in Mp4071. Fifty-eight SCR effector proteins were identified in MrPeru, 49 in Mp4145, 58 in Mp1441, 33 in Mp4124, 28 in Mp178 and 26 in Mp4071. The prediction of RCP varied from 79 (MrPeru) to 23 (Mp4017) proteins. In total, the EffectorP predicted 164 effector candidates in MrPeru, 105 in Mp4145, 102 in Mp1441, 71 in Mp4124, 57 in Mp178 and 60 in Mp4071 (Fig. 1).

In the final predicted CSEPs dataset, very few proteins were predicted with two of the three criteria considered for the prediction of effectors (NLS, SCR and RCP), and none of the proteins presented the three criteria. Mostly, putative effector candidates showed only one of these characteristics. EffectorP also found most of the CSEPs of SCR type. In addition, EffectorP also predicted CSEPs that were not present in any of the three criteria described above (Additional file 4: Figure S1). The total arsenal of CSEPs from all the isolates (effectorome) was obtained by taking all the sequences that obeyed the three criteria used in the pipeline (NLS, SCR and RCP) plus those predicted by EffectorP. The individual repertoire of predicted CSEPs were 243 for MrPeru, 157 for Mp4145, 134 for Mp1441, 109 for Mp4124, 92 for Mp178 and 80 for Mp4071 (Fig. 1). The effector lists are available in Additional file 5: Table S4, separated by category (NLS, SCR, RCP and those predicted by the EffectorP).

### Functional characterization of CESPs

**Fig. 3:**
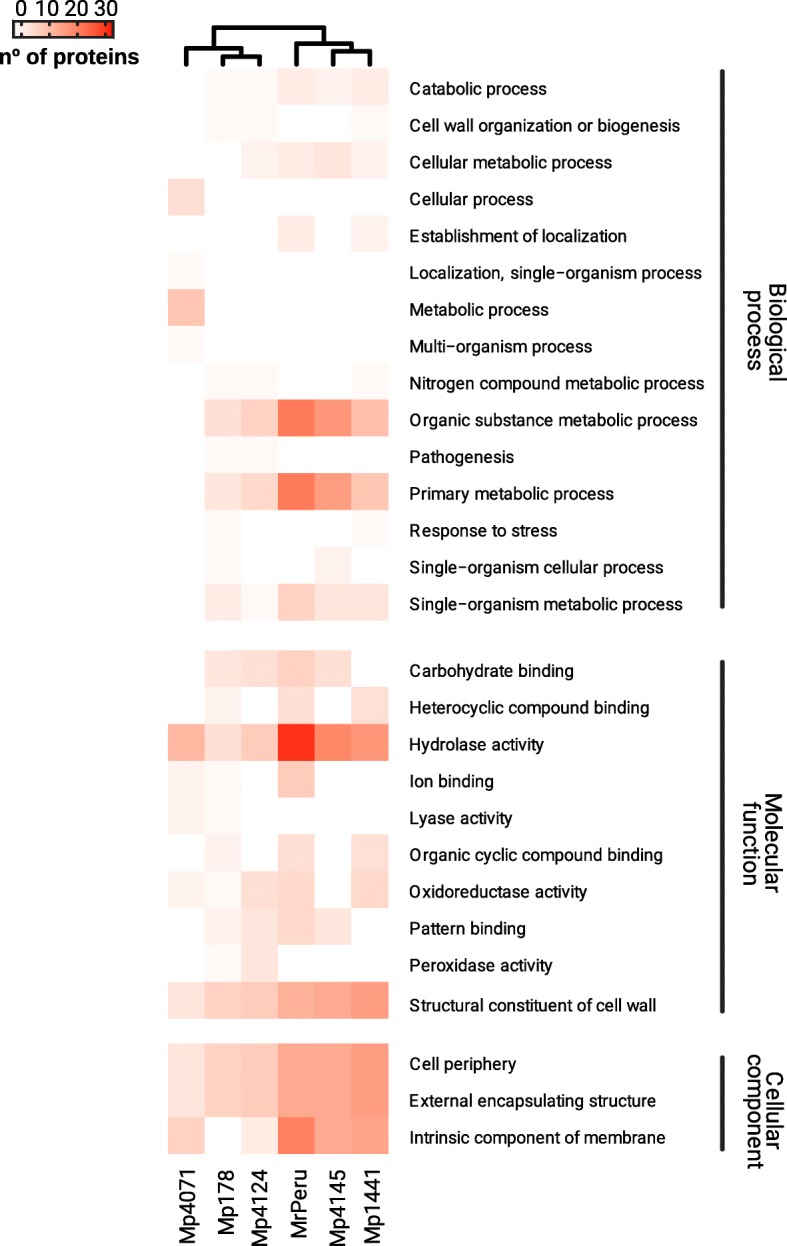
Functional characterization hitmap of the CSEPs with the blast2GO results. The Functional characterization hitmap of the CSEPs with the blast2GO results used Level 3 Gene Ontology hierarchy for biological processes, molecular function and cellular component. MrPeru - *Moniliophthora roreri* isolate from Peruvian subpopulation. Mp4145 and Mp1441- *M. perniciosa* isolates from cacao subpopulations in Bahia. Mp4124 - *M. perniciosa* isolate from cacao subpopulation in Ecuador. Mp178 - *M. perniciosa* isolate from wild solanaceous subpopulation (Lobeira) in Minas Gerais. Mp4071 - *M. perniciosa* isolate from wild solanaceous subpopulation (Caiçara) in Bahia

The putative functional characterization of CESPs performed with BLAST2GO were separated according to the biological processes, molecular function and cellular component in which they are involved (Fig. 3, Additional file 6: Table S5).

#### Biological processes

The identified CSEPs were separated according to the biological processes in which they are involved. In MrPeru, 56 proteins were related to biological processes, 43 in Mp4145, 33 in Mp1441, 20 in Mp4124, 18 in Mp178 and Mp4071. Among the biological processes, organic substance metabolic processes and primary metabolic processes showed a higher number of proteins with these functions in MrPeru, Mp4145, Mp1441, Mp4124 and Mp178. Mp4071 showed more proteins with functions in metabolic processes and cellular processes. Pathogenesis function, despite in smaller amounts, was found in Mp178 and Mp4124. Other biological processes have the function of establishment (MrPeru and Mp1441), cell wall organization or biogenesis (Mp1441, Mp4071 and Mp178) and response to stress (Mp1441 and Mp178), which may be related to the plant-pathogen interaction. A large number of families of glycoside hydrolases were found in all isolates. Endoglucanase II was also identified in four of the isolates (MrPeru, Mp4145, Mp4124 and Mp178), except for Mp1441 and Mp4071. Carbohydrate esterase families were detected in all isolates, except Mp178.

#### Molecular function

In general, 77 proteins were identified in MrPeru, 40 in Mp4145, 47 in Mp1441, 34 in Mp4124, 26 in Mp178 and 21 in Mp4071 related to molecular functions. Among them, hydrolase activity and structural constituent of cell wall were the most frequent and common to all isolates. In addition to hydrolase activity, other enzymes with lyase (in Mp178 and Mp4071), oxidoreductase (MrPeru, Mp1441, Mp4124, Mp178 and Mp4071) and peroxidase (Mp178 and Mp4124) activities were identified.

#### Cell component

In the cell component category, 45 proteins were identified in MrPeru, 39 in Mp4145, 44 in Mp1441, 19 in Mp4124, 14 in Mp178 and 15 in Mp4071. The three functions that stood out were cell periphery, external encapsulating structures and intrinsic components of membranes in all isolates. The latter function was not found in Mp178.

### Core effectors

**Fig. 4:**
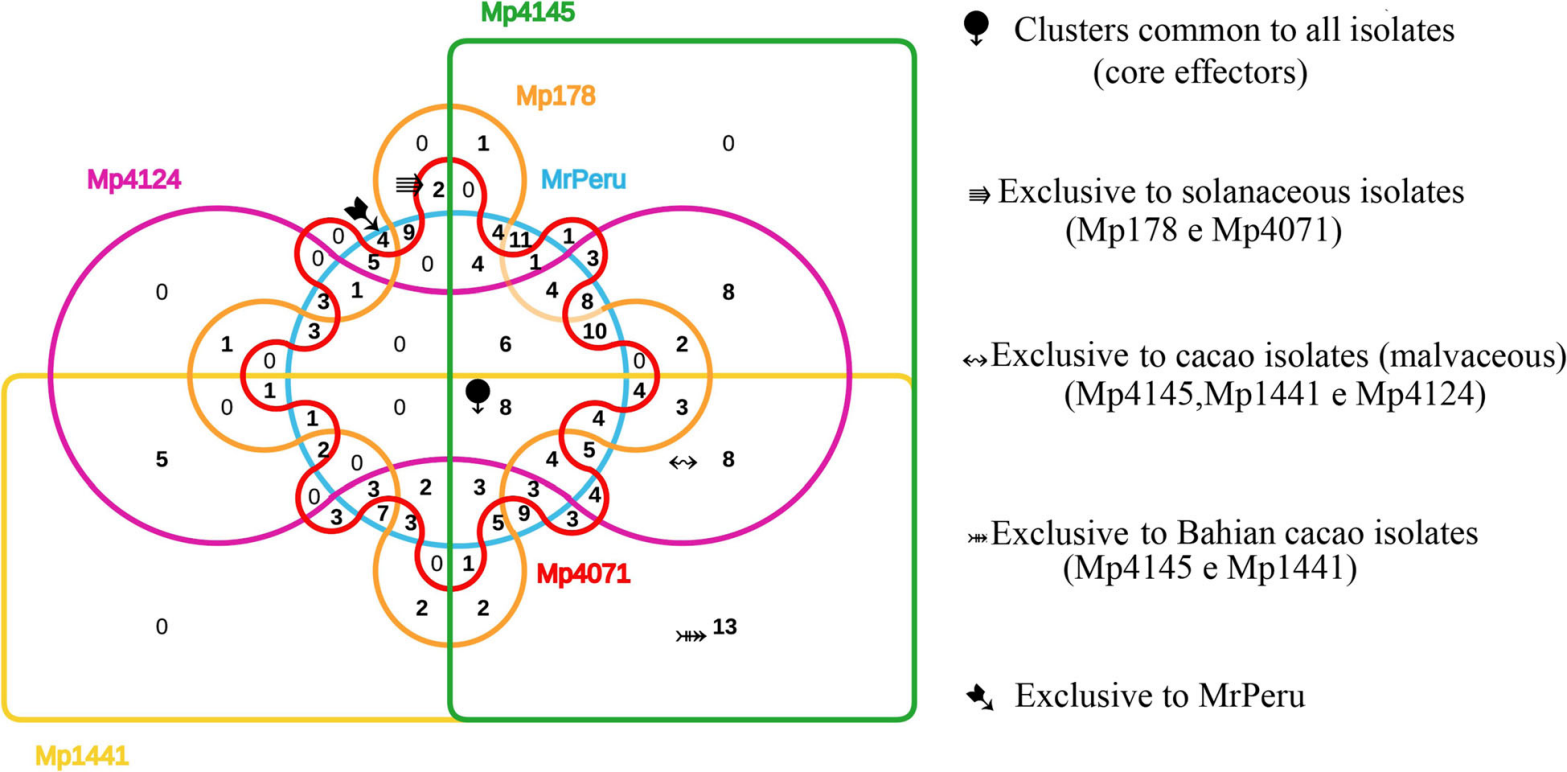
Distribution and clustering of CSEPs repertoire among six *Moniliophthora* genomes. The number of proteins shared among isolates are indicated: eight clusters with 49 proteins were common to all isolates, four clusters were exclusive to MrPeru, eight clusters with a total of 24 proteins exclusives to cacao isolates (Mp4145, Mp1441 and Mp4124), two clusters with four proteins exclusive to solanaceous isolates (Mp178 and Mp4071). Thirteen clusters with 26 proteins were exclusive to Bahian isolates from cacao (Mp1441 and Mp4145). MrPeru - *Moniliophthora roreri* isolate from Peruvian subpopulation. Mp4145 and Mp1441- *M. perniciosa* isolates from cacao subpopulations in Bahia. Mp4124 - *M. perniciosa* isolate from cacao subpopulation in Ecuador. Mp178 - *M. perniciosa* isolate from wild solanaceous subpopulation (Lobeira) in Minas Gerais. Mp4071 - *M. perniciosa* isolate from wild solanaceous subpopulation (Caiçara) in Bahia

We used the OrthoVenn [45] to identify orthologous genome clusters among the CSEPs of the six isolates; the sequence similarity was calculated with e-value cut-off of 1e-25 and inflation value of 2.5. The Venn diagram represents orthologous clusters among the sequences. The diagram pointed out eight clusters shared among isolates (Fig. 4, Additional file 7: Table S6A), with a total of 49 CSEPs. Only two of the clusters showed functional annotation. Of the two annotated clusters, one presented cell component function acting in the extracellular region, and the other with GO for cellular component: actin cortical patch, endosome and plasma membrane; molecular function: calcium ion binding and biological process: endocytosis, with hit against the Swiss-Prot for Protein SnodProt1 and actin cytoskeleton-regulatory complex protein PAN1. These clusters were considered as core effectors of the *Moniliophthora* genus, suggesting that conserved genes are involved in the pathogenicity of these fungi.

### Exclusive effectors

We found four unique clusters in MrPeru with eight proteins, of which only one cluster was annotated, with homology to the cell wall protein DAN4 (Fig. 4, Additional file 7: Table S6B). The other isolates did not show exclusive clusters for each individual; however, there were exclusive clusters to a host subpopulation. Eight clusters were shared among cacao isolates (Mp4145, Mp1441 and Mp4124) with a total of 24 proteins, which showed two annotated clusters with homology against the Swiss-Prot database for fruiting body protein SC3 and fruiting body protein SC7. These proteins are structural constituent of the cell wall that operates in the extracellular region (Fig. 4, Additional file 7: Table S6C). Thirteen clusters were exclusive of the Bahian isolates from cacao host (Mp1441 and Mp4145), with homology for four of them: endoglucanase-1, hyphally regulated protein, fruiting body protein SC3 and pheromone-processing carboxypeptidase KEX1 (Fig. 4, Additional file 7: Table S6D). Two clusters were exclusive to the solanaceous isolates (Mp178 and Mp4071), one with homology to a hyphally regulated cell wall protein 3, with GO for cellular component: anchored component of membrane, cell surface, extracellular region and fungal-type cell wall; and biological processes involved with pathogenesis (Fig. 4, Additional file 7: Table S6E).

Sequences that were not clustered by OrthoVenn were grouped into singletons. One-hundred and three singletons were identified in MrPeru, 15 in Mp4145, 26 in Mp1441, 10 in Mp4124, 11 in Mp178 and 14 in Mp4071 sequences (Table 2). The list of singletons is available in Additional file 7: Table S6.

**Table 2:** Summary OrthoVenn

	CSEPs	Clusters	Singletons
MrPeru	243	131	103
Mp4145	157	141	15
Mp1441	134	108	26
Mp4124	109	98	10
Mp178	92	81	11
Mp4071	80	66	14

Summary of OrthoVenn with total CSEPs, orthologous clusters (at least contains two species) and singletons. MrPeru - *Moniliophthora roreri* isolate from Peruvian subpopulation. Mp4145 and Mp1441 - *M. perniciosa* isolates from cacao subpopulations in Bahia. Mp4124 - *M. perniciosa* isolate from cacao subpopulation in Ecuador. Mp178 - *M. perniciosa* isolate from wild solanaceous subpopulation (Lobeira) in Minas Gerais. Mp4071 - *M. perniciosa* isolate from wild solanaceous subpopulation (Caiçara) in Bahia

## Discussion

Within the genus *Moniliophthora*, the main notorious plant pathogens are *M. perniciosa* and *M. roreri* because they are the causal agents of the most important diseases on cacao – the chocolate tree – in the Americas. The dissemination of these pathogens most likely spread alongside with the propagation of cacao cultivation. The release of the sequencing of the genomes of *M. perniciosa* [16] (genome size 26.66 Mb) and *M. roreri* [4] (genome size 52.3 Mb) plant pathogens along with the *T. cacao* genome, their host plant [46, 47], represent a significant milestone in the era of “genomics”.

*Moniliophthora perniciosa* can infect more than five species, both horticultural and wild solanaceas, which is a rather unusual feature for this fungus that is highly efficient to cause disease in cacao [21, 48]. In contrast, *M. roreri* is a highly specialized pathogen of cacao plants, infecting only pods. Within *M. roreri*, genetic diversity studies have indicated the occurrence of five genetically diverse groups [19]. The isolate used herein is from Peru, a representative of the Bolivar group, which comprises isolates from Peru, Colombia, Venezuela and Ecuador. In this work, we generated a assembly and annotation of the genome of *M. perniciosa* isolates/subpopulation that varies for pathogenicity to cacao genotypes, and a *M. roreri* isolate representative of one of the major *M. roreri* group.

The genome sizes differ from those reported in literature [11, 16]. MrPeru was somewhat smaller (45.17 Mb) than the total genome of *M. roreri* described by Meinhardt et al. [11] who reported a size of 52.3 Mb. The genome sizes of *M. perniciosa* isolates was estimated to be higher, between 47.01 and 43.92 Mb, than that described by Mondego et al. [16]. These differences are expected, and presumably are attributable to the isolates used herein or to assembly strategies. Our results are supported with the profiles found in the species and in agreement with predicted number of genes, as well as in accordance with the results in other fungi. Although long-read sequencing in genomics platforms and/or RNAseq data could be used to further look deep into the genome, our data allowed gaining insight into the potential repertoire of small secreted proteins (effectors) of *M. perniciosa* and *M. roreri* pathogens.

Our assemblies of *M. perniciosa* and *M. roreri* isolates allowed us to identify phylogenetic relationships and CSEPs molecules of *Moniliophthora*, which are key for a successful host infection and pathogenic adaptation. This knowledge will be used to develop strategies aiming to limit the spread of WB and FPR. We used a conservative approach to predict the array of effectors, and so we believe we are presenting a representative set of CSEPs for these isolates.

Inferred phylogeny was consistent with the previous studies using *M. perniciosa* from Solanaceous and Malvaceous isolates, pointing to a common ancestor and sustained the relationship among host subpopulations [9]. Hence, these isolates are expected to share more homologues among them and show similar expansion or contraction of certain gene families.

On average, about 32% of the effectors found in the isolates showed to be small proteins rich in cysteine. Although mostly of the SCRs are related to apoplastic effectors, there are SCR effectors that can act on the cytoplasm as well such as the AvrP4 and AvrP123 effectors of *Melampsora lini* that are recognized by intracellular immune receptors [49]. GO analysis showed that most of the CSEPs are likely to respond to oxidative stress. These proteins may be secreted to counteract host generated oxidative stress.

CSEPs that are RCP corresponded on average to 30.5% of the effectorome of the isolates. This is important because some effectors are characterized by being in unstable regions in the genome, as in repeat-rich regions and centromeres, which may be fully connected with their high polymorphic potential. This high polymorphism that characterizes effectors can promote their evolution, an important factor for pathogen adaptation and avoidance of the plant immune system, thus allowing a successful infection process [43].

The first line of plant defense is the recognition of pathogen associated molecular pattern (PAMPs), thus activating plant immune system triggering effector-induced immunity (ETI) [50]. In this line, we found an abundance of proteins associated with cell periphery, external encapsulating structure, and intrinsic component of membrane and structural constituent of cell wall compounding an arsenal of proteins that may act as putative effectors that might limit the entry of microbes, restrict fungal colonization or kill pathogens within the host plant.

Functional characterization of the effector candidates is consistent with the results of Ferreira [51], who observed that the secreted protein profile of *M. perniciosa* of cacao and solanaceous hosts consists of an enzymatic arsenal, resulting in effector-triggered susceptibility (ETS). Among these enzymes we found a great amount of hydrolases [50]. Presence of hydrolases in the secretome of other pathogens has been associated with the degradation of polymers of the plant cell wall, allowing fungal penetration into host tissues, besides being a source of water and nutrients for them [50]. For ex, in *Aspergillus flavus* the production of extracellular hydrolases was linked to its survival on a variety of substrates and penetration into host tissues [52]. Also, Meinhardt et al. [4] analysis of *M. roreri* transcriptome revealed 11 differentially expressed glycoside hydrolases in the biotrophic phase of the *M. roreri*. We propose that these proteins, potentially, allow the pathogen to degrade plant compounds and initiate infection even in the presence of the high oxidative stress environment, but it is evident that additional study is required to test this hypothesis.

The overrepresented GO categories associated with biological processes were those related to energy metabolism, especially metabolism of compounds involved with carbohydrates. CSEPs were lipases, hydrophobins and necrosis-inducing endopolygalacturonases nature. These results suggested that *M. perniciosa* secretome consists of diverse proteins that function in an organized manner to suppress different aspects of fungal colonization for disease success [8]. Ferreira [51] also described this type of proteins in their work, and related them with important roles in several biological process, pathophysiological processes.

The determination of the core effectors, either to the genus or each subpopulation, suggests that these putative effectors are highly conserved and are essential proteins for pathogenicity, being non-specific for infection on the different hosts [38] and probably specific to these pathosystems. In contrast, the unique CSEPS of each species/subpopulation/isolate may be involved with the specificity with which they infect and how they infect each host.

## Conclusion

The repertoire of plant pathogen effectors is key to understanding the plant-pathogen interaction and the co-evolution process of the pathosystem. The present work provided a database of the putative effectorome of *Moniliophthora* isolates and species. Of further interests is the identified set of core effector conserved in all isolates. This is an important finding as it is expected to be related with the adaptation of different lineages to different hosts. Inevitably, this finding opens numerous new questions about the biology of these fungi. Thus, the current set of effectors found in *M. roreri* and *M. perniciosa* are valuable resources for future studies of effector function and evolution of these plant pathogens. In addition, they can be used as tools to search for cacao defense against these plant pathogens aiming to achieve plants with durable resistance.

## Additional files

Additional file 1: **Table S1.** Bioinformatics tools used to predict CSEPs. (DOCX 9 kb)

Additional file 2: **Table S2.** Assessment of genome quality by BUSCO. (DOCX 12 kb)

Additional file 3: **Table S3.** Repeat elements in the genomes. (DOCX 14 kb)

Additional file 4: **Figure S1.** Venn diagrams: comparison of results of the effectors: (I) Nuclear Location Signal (NLS), (II) small and cysteine rich (SCR), and (III) repeats containing protein (RCP) and effectors predicted by EffectorP. (DOCX 759 kb)

Additional file 5: **Table S4.** Lists of CSEPs. (XLSX 26 kb)

Additional file 6: **Table S5.** Annotation of the CSEPs. (XLSX 50 kb)

Additional file 7: **Table S6.** List of common and unique clusters and singletons. (XLSX 16 kb)

## Abbreviations

BUSCO: Benchmarking Universal Single-Copy Orthologs
CBG: Center of Biotechnology and Genetics
CSEPs: Candidate secreted effector proteins
ETI: Effector-triggered immunity
ETS: Effector-triggered susceptibility
FPR: Frosty pod rot
GO: Gene Ontology
ML: Maximum likelihood
Mp1441: *Moniliophthora perniciosa*, from cacao, Bahian subpopulation (2003)
Mp178: *Moniliophthora perniciosa*, solanaceous hosts (wild solanaceous – Lobeira), from Minas Gerais
Mp4071: *Moniliophthora perniciosa*, solanaceous hosts (wild solanaceous – Caiçara), from Bahia
Mp4124: *Moniliophthora perniciosa*, from cacao, Ecuadorian subpopulation
Mp4145: *Moniliophthora perniciosa*, from cacao, Bahian subpopulation (2012)
MrPeru: *Moniliophthora roreri*, from Peruvian subpopulation Peru
NLS: Nuclear localization signal
RCP: Repeats containing protein
SCR: Small and cysteine rich
TM: Transmembrane domain
WB: Witches’ broom

## References

[1] Aime MC, Phillips-Mora W (2005). The causal agents of witches’ broom and frosty pod rot of cacao (chocolate, *Theobroma cacao*) form a new lineage of Marasmiaceae. Mycologia.

[2] Meinhardt LW, Rincones J, Bailey BA, Aime MC, Griffith GW, Zhang D (2008). *Moniliophthora perniciosa*, the causal agent of witches’ broom disease of cacao: What’s new from this old foe?. Mol Plant Pathol.

[3] Phillips-Mora W, Coutiño A, Ortiz CF, López AP, Hernández J, Aime MC (2006). First report of *Moniliophthora roreri* causing frosty pod rot (moniliasis disease) of cocoa in Mexico. Plant Pathol.

[4] Meinhardt LW, Costa GGL, Thomazella DPT, Teixeira PJPL, Carazzolle MF, Schuster SC (2014). Genome and secretome analysis of the hemibiotrophic fungal pathogen, *Moniliophthora roreri*, which causes frosty pod rot disease of cacao: mechanisms of the biotrophic and necrotrophic phases. BMC Genomics.

[5] Bailey BA, Evans HC, Phillips-Mora W, Ali SS, Meinhardt LW. *Moniliophthora roreri*, causal agent of cacao frosty pod rot. Mol Plant Pathol. 2018; 10.1111/mpp.12648. 10.1111/mpp.12648 PMC6638017 29194910

[6] Griffith GW, Nicholson J, Nenninger A, Birch RN, Hedger JN (2003). Witches’ brooms and frosty pods: two major pathogens of cacao. New Zeal J Bot.

[7] Griffith GW, Hedger JN (1994). The breeding biology of biotypes of the witches’ broom pathogen of cocoa, *Crinipellis perniciosa*. Heredity (Edinb).

[8] Rincones J, Mazotti GD, Griffith GW, Pomela A, Figueira A, Leal GA (2006). Genetic variability and chromosome-length polymorphisms of the witches’ broom pathogen *Crinipellis perniciosa* from various plant hosts in South America. Mycol Res.

[9] Patrocínio NGRB. Genética e evolução de *Moniliophthora perniciosa* de hospedeiros solanáceos e malváceos. Universidade Estadual de Santa Cruz, Brazil. Genet Mol Biol. 2016;

[10] Gramacho KP, Newman Luz EDM, Da Silva FS, Lopes UV, Pires JL, Pereira L (2016). Pathogenic variability of *Moniliophthora perniciosa* in three agroecological zones of the cacao region of Bahia, Brazil. Crop Breed Appl Biotechnol.

[11] Dalio RJD, Magalhães DM, Atílio LB, Rodrigues CM, Breton MC, Pichi S, Pascholati SF, Machado MA. Efetores nas Interações Planta-patógeno. Revisao Anu Patol Plantas. 2014:1–44.

[12] Ellis JG, Rafiqi M, Gan P, Chakrabarti A, Dodds PN (2009). Recent progress in discovery and functional analysis of effector proteins of fungal and oomycete plant pathogens. Curr Opin Plant Biol.

[13] Hacquard S, Joly DL, Lin Y-C, Tisserant E, Feau N, Delaruelle C (2012). A comprehensive analysis of genes encoding small secreted proteins identifies candidate effectors in *Melampsora larici-populina* (poplar leaf rust). Mol Plant-Microbe Interact.

[14] Park J, Zhang Y, Buboltz AM, Zhang X, Schuster SC, Ahuja U (2012). Comparative genomics of the classical Bordetella subspecies: the evolution and exchange of virulence-associated diversity amongst closely related pathogens. BMC Genomics.

[15] Mondego JMC, Carazzolle MF, Costa GGL, Formighieri EF, Parizzi LP, Rincones J (2008). A genome survey of *Moniliophthora perniciosa* gives new insights into witches’ broom disease of cacao. BMC Genomics.

[16] Mondego JM, Carazzolle MF, Costa GG, Formighieri EF, Parizzi LP, Rincones J (2008). A genome survey of *Moniliophthora perniciosa* gives new insights into witches’ broom disease of cacao. BMC Genomics.

[17] Rincones J, Scarpari LM, Carazzolle MF, Mondego JMC, Formighieri EF, Barau JG (2008). Differential gene expression between the biotrophic-like and saprotrophic mycelia of the witches’ broom pathogen *Moniliophthora perniciosa*. Mol Plant-Microbe Interact.

[18] Micheli F, Guiltinan M, Gramacho KP, Wilkinson MJ, Figueira VDO, Maximova S, Delseny M (2010). Functional genomics of cacao. Adv Bot Res.

[19] Phillips-Mora W, Aime MC, Wilkinson MJ (2007). Biodiversity and biogeography of the cacao (Theobroma cacao) pathogen *Moniliophthora roreri* in tropical America. Plant Pathol.

[20] Jucá Santos FF, Vanderlei Lopes U, Pires JL, Pires Melo GR, Peres Gramacho K, Clément D (2014). QTLs detection under natural infection of *Moniliophtora perniciosa* in a cacao F_2_ progeny with scavina-6 descendants. Agrotrópica (Itabuna).

[21] Patrocínio NGRB, Ceresini PC, Gomes LIS, Resende MLV, Mizubuti ESG, Gramacho KP (2017). Population structure and migration of the witches’ broom pathogen *Moniliophthora perniciosa* from cacao and cultivated and wild solanaceous hosts in southeastern Brazil. Plant Pathol.

[22] H KS, Gramacho KP, Lopes UV. Genetic Diversity of *Moniliophthora perniciosa* Populations from Cocoa in Ecuador. 2005. p. 85–99. CFC Technical Paper N 55.

[23] Castellani A (1967). Maintenance and cultivation of the common pathogenic fungi of man in sterile distilled water. Further researches. J trop Med Hyg.

[24] White TJ, Bruns T, Lee S, Taylor J. Amplification and Direct Sequencing of Fungal Ribosomal RNA Genes for Phylogenetics. PCR Protoc. 1990:315–22.

[25] Tarailo-Graovac M, Chen N. Using RepeatMasker to identify repetitive elements in genomic sequences. Curr Protoc Bioinformatics. 2009:1–14. 10.1002/0471250953.bi0410s25 19274634

[26] Simão FA, Waterhouse RM, Ioannidis P, Kriventseva EV, Zdobnov EM (2015). BUSCO: assessing genome assembly and annotation completeness with single-copy orthologs. Bioinformatics.

[27] Stanke M, Keller O, Gunduz I, Hayes A, Waack S, Morgenstern B (2006). AUGUSTUS: a b initio prediction of alternative transcripts. Nucleic Acids Res.

[28] Cantarel BL, Korf I, Robb SMC, Parra G, Ross E, Moore B (2008). MAKER: an easy-to-use annotation pipeline designed for emerging model organism genomes. Genome Res.

[29] Edgar RC (2004). MUSCLE: multiple sequence alignment with high accuracy and high throughput. Nucleic Acids Res.

[30] Stamatakis A (2014). RAxML version 8: a tool for phylogenetic analysis and post-analysis of large phylogenies. Bioinformatics.

[31] Letunic I, Bork P (2016). Interactive tree of life (iTOL) v3: an online tool for the display and annotation of phylogenetic and other trees. Nucleic Acids Res.

[32] Petersen TN, Brunak S, Von Heijne G, Nielsen H (2011). SignalP 4.0: discriminating signal peptides from transmembrane regions. Nat Methods.

[33] Käll L, Krogh A, Sonnhammer ELL (2007). Advantages of combined transmembrane topology and signal peptide prediction-the Phobius web server. Nucleic Acids Res.

[34] Hiller K, Grote A, Scheer M, Münch R, Jahn D (2004). PrediSi: prediction of signal peptides and their cleavage positions. Nucleic Acids Res.

[35] Emanuelsson O, Nielsen H, Brunak S, Von Heijne G (2000). Predicting subcellular localization of proteins based on their N-terminal amino acid sequence. J Mol Biol.

[36] Briesemeister S, Blum T, Brady S, Lam Y, Kohlbacher O, Shatkay H (2009). SherLoc2: a high-accuracy hybrid method for predicting subcellular localization of proteins. J Proteome Res.

[37] Krogh A, Larsson B, Von Heijne G, Sonnhammer ELL (2001). Predicting transmembrane protein topology with a hidden Markov model: application to complete genomes. J Mol Biol.

[38] Toro KD, Brachmann A (2016). The effector candidate repertoire of the arbuscular mycorrhizal fungus *Rhizophagus clarus*. BMC Genomics.

[39] Nguyen Ba AN, Pogoutse A, Provart N, Moses AM (2009). NLStradamus: a simple hidden Markov model for nuclear localization signal prediction. BMC Bioinform.

[40] Newman AM, Cooper JB (2007). XSTREAM: A practical algorithm for identification and architecture modeling of tandem repeats in protein sequences. BMC Bioinform.

[41] Sperschneider J, Gardiner DM, Dodds PN, Tini F, Covarelli L, Singh KB (2016). EffectorP: predicting fungal effector proteins from secretomes using machine learning. New Phytol.

[42] Conesa A, Götz S, García-Gómez JM, Terol J, Talón M, Robles M (2005). Blast2GO: a universal tool for annotation, visualization and analysis in functional genomics research. Bioinformatics.

[43] Saunders DGO, Win J, Cano LM, Szabo LJ, Kamoun S, Raffaele S (2012). Using hierarchical clustering of secreted protein families to classify and rank candidate effectors of rust fungi. PLoS One.

[44] Kamoun S (2006). A catalogue of the effector secretome of plant pathogenic oomycetes. Annu Rev Phytopathol.

[45] Wang Y, Coleman-Derr D, Chen G, Gu YQ (2015). OrthoVenn: a web server for genome wide comparison and annotation of orthologous clusters across multiple species. Nucleic Acids Res.

[46] Argout X, Salse J, Aury JM, Guiltinan MJ, Droc G, Gouzy J (2011). The genome of *Theobroma cacao*. Nat Genet.

[47] Motamayor JC, Mockaitis K, Schmutz J, Haiminen N, Livingstone D, Cornejo O (2013). The genome sequence of the most widely cultivated cacao type and its use to identify candidate genes regulating pod color. Genome Biol.

[48] Lopes JRM, Luz EDMN, Bezerra JL. Suscetibilidade do cupuaçuzeiro e outras espécies vegetais a isolados de *crinipellis perniciosa* obtidos de quatro hospedeiros diferentes no sul da Bahia. 2001;26:601–5.

[49] Sperschneider J, Dodds PN, Gardiner DM, Manners JM, Singh KB, Taylor JM (2015). Advances and challenges in computational prediction of effectors from plant pathogenic fungi. PLoS Pathog.

[50] Hématy K, Cherk C, Somerville S (2009). Host-pathogen warfare at the plant cell wall. Curr Opin Plant Biol.

[51] Ferreira RDM. Componentes epidemiológicos e perfil de proteínas secretadas de isolados de *Moniliophthora perniciosa *de cacaueiro e hospedeiros solanáceos. Ilheus: Universidade Estadual de Santa Cruz, Brazil; 2016. p. 99. http://nbcgib.uesc.br/genetica/admin/images/files/rafaella_ferreira.pdf.

[52] Mellon JE, Cotty PJ, Dowd MK (2007). *Aspergillus flavus* hydrolases: their roles in pathogenesis and substrate utilization. Appl Microbiol Biotechnol.

