# Supplementary material for: Genome sequence and effectorome of Moniliophthora perniciosa and Moniliophthora roreri subpopulations
Source: BMC Genomics. 2018 Jul 3;19:509. doi: 10.1186/s12864-018-4875-7 (PMC6029071; doi:10.1186/s12864-018-4875-7)
Supplement: Supplementary file 1 — Table S1. Bioinformatics tools used to predict CSEPs. (DOCX 9 kb) [file 12864_2018_4875_MOESM1_ESM.docx]

**Additional file 1: table S1****.** Bioinformatics tools used to predict CSEPs.

| **Tool** | **Prediction** | **Website** |
| --- | --- | --- |
| ***Secretome*** |  |  |
| SignalP | Presence and location of signal peptide | <http://www.cbs.dtu.dk/services/SignalP/> |
| Phobius | Transmembrane topology and signal peptide | <http://phobius.sbc.su.se/> |
| PrediSi | Signal peptide | <http://www.predisi.de/> |
| TargetP | Subcellular location of eukaryotic proteins | <http://www.cbs.dtu.dk/services/TargetP/> |
| SherLoc2 | Proteins subcellular location | <http://www-bs.informatik.uni-tuebingen.de/Services/SherLoc2> |
| ***Effectorome*** |  |  |
| TMHMM | Transmembrane helices in proteins | <http://www.cbs.dtu.dk/services/TMHMM/> |
| NLStradamos | Nuclear localization signals (NLSs) | <http://www.moseslab.csb.utoronto.ca/NLStradamus> |
| XTREAM | Identify tandem repeat (TR) | <http://jimcooperlab.mcdb.ucsb.edu/xstream/> |
| EffectorP | predicting fungal effector proteins from secretome using machine learning | <http://effectorp.csiro.au/> |
