# Supplementary material for: Genome sequence and effectorome of Moniliophthora perniciosa and Moniliophthora roreri subpopulations
Source: BMC Genomics. 2018 Jul 3;19:509. doi: 10.1186/s12864-018-4875-7 (PMC6029071; doi:10.1186/s12864-018-4875-7)
Supplement: Supplementary file 2 — Table S2. Assessment of genome quality by BUSCO. (DOCX 12 kb) [file 12864_2018_4875_MOESM2_ESM.docx]

**Additional file 2: Table S2** Assessment of genomes quality by BUSCO

|  | **MrPeru** | **Mp4145** | **Mp1441** | **Mp4124** | **Mp178** | **Mp4071** |
| --- | --- | --- | --- | --- | --- | --- |
| Complete BUSCOs | 1281  (95,9%) | 1187  (88,9%) | 881  (66%) | 991  (74.3%) | 1101  (82.5%) | 919  (68.8%) |
| Complete and single-copy  BUSCOs | 1267 | 1171 | 874 | 982 | 1092 | 913 |
| Complete and duplicated BUSCOs | 14 | 16 | 7 | 9 | 9 | 6 |
| Fragmented BUSCOs | 27 | 74 | 218 | 159 | 105 | 190 |
| Missing BUSCOs | 27 | 74 | 236 | 185 | 129 | 226 |
|  |  |  |  |  |  |  |
