# Supplementary material for: Genome sequence and effectorome of Moniliophthora perniciosa and Moniliophthora roreri subpopulations
Source: BMC Genomics. 2018 Jul 3;19:509. doi: 10.1186/s12864-018-4875-7 (PMC6029071; doi:10.1186/s12864-018-4875-7)
Supplement: Supplementary file 3 — Table S3. Repeat elements in the genomes. (DOCX 14 kb) [file 12864_2018_4875_MOESM3_ESM.docx]

**Additional file 3: Table S3.** Repeat elements in the genomes

|  | **Types of elements** | **Number of elements** | **Size of elements (bp)** | **Percentage in the genome (%)** |
| --- | --- | --- | --- | --- |
| **MrPeru** | LTRs  Ty1/Copia  Gypsy/DIRS1  DNA transposons  Unclassified  Small RNA  Simple repeats  Low complexity | 794  145  627  79  22  12  3983  571 | 361565  55116  298148  10826  3748  16598  159829  28334 | 0.80  0.12  0.66  0.02  0.01  0,04  0.35  0.06 |
| **Mp4145** | LTRs  Ty1/Copia  Gypsy/DIRS1  DNA transposons  Unclassified  Small RNA  Simple repeats  Low complexity | 811  118  677  98  18  5  4091  597 | 447620  42671  397134  15878  3369  5533  166256  29316 | 0.95  0.09  0.84  0.03  0.01  0.01  0.35  0.06 |
| **Mp1441** | LTRs  Ty1/Copia  Gypsy/DIRS1  DNA transposons  Unclassified  Small RNA  Simple repeats  Low complexity | 581  99  470  81  18  5  3392  532 | 285617  34073  245863  11131  2443  5533  137431  26019 | 0.62  0.07  0.53  0.02  0.01  0.01  0.30  0.06 |
| **Mp4124** | LTRs  Ty1/Copia  Gypsy/DIRS1  DNA transposons  Unclassified  Small RNA  Simple repeats  Low complexity | 536  89  435  61  16  4  3202  515 | 257001  26975  224641  9078  2570  5417  129968  25831 | 0.56  0.06  0.49  0.02  0.01  0.01  0.29  0.06 |
| **Mp178** | LTRs  Ty1/Copia  Gypsy/DIRS1  DNA transposons  Unclassified  Small RNA  Simple repeats  Low complexity | 350  57  286  45  14  4  2765  414 | 146879  20087  123147  8502  2804  5307  110179  20209 | 0.33  0.05  0.28  0,02  0.01  0.01  0.25  0.05 |
| **Mp4071** | LTRs  Ty1/Copia  Gypsy/DIRS1  DNA transposons  Unclassified  Small RNA  Simple repeats  Low complexity | 405  79  317  53  12  4  2753  436 | 173512  28438  141217  7012  1886  5307  110978  21416 | 0.39  0.06  0.32  0.02  0.00  0.01  0.25  0.05 |
