# Supplementary material for: Genome sequence and effectorome of Moniliophthora perniciosa and Moniliophthora roreri subpopulations
Source: BMC Genomics. 2018 Jul 3;19:509. doi: 10.1186/s12864-018-4875-7 (PMC6029071; doi:10.1186/s12864-018-4875-7)
Supplement: Supplementary file 4 — Figure S1. Venn diagrams: comparison of results of the effectors: (I) Nuclear Location Signal (NLS), (II) small and cysteine rich (SCR), and (III) repeats containing protein (RCP) and effectors predicted by EffectorP. (DOCX 759 kb) [file 12864_2018_4875_MOESM4_ESM.docx]

**Additional file 6: Figure S1.** Venn diagrams comparing the results of the effectors

**MrPeru Mp4145**


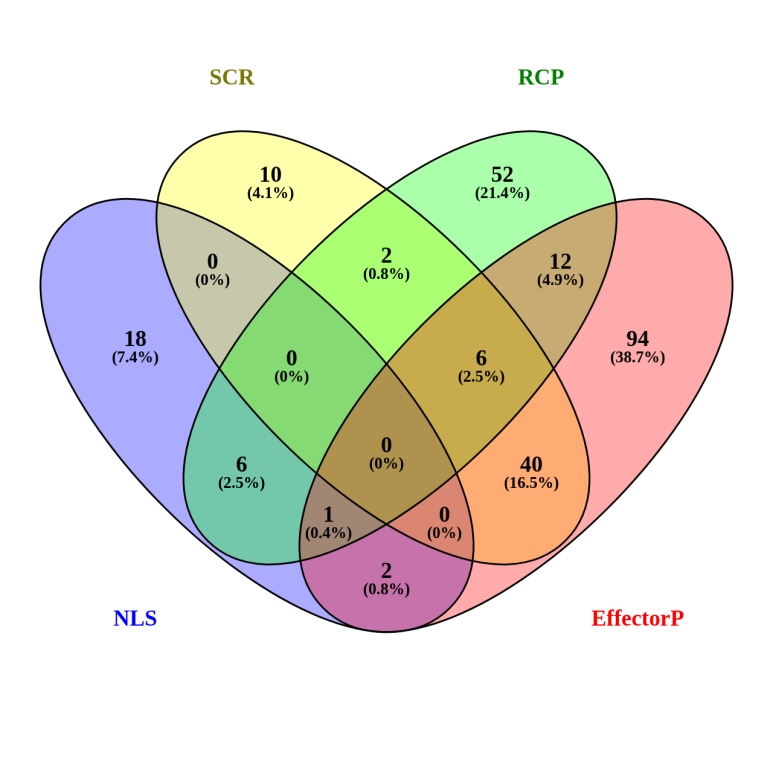

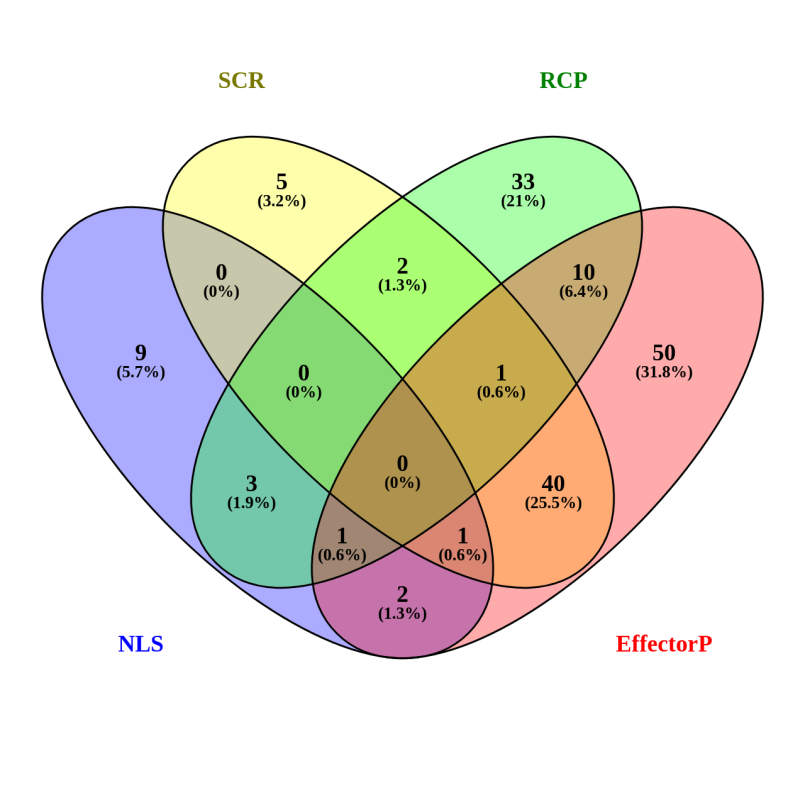


**Mp1441 Mp4124**

**
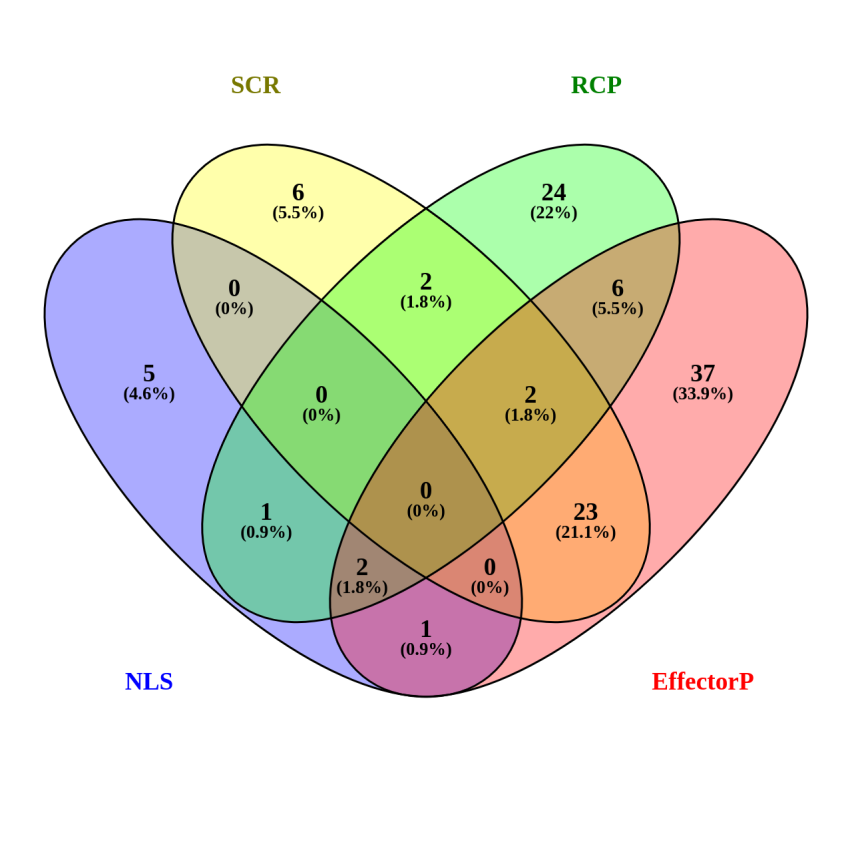
**

**
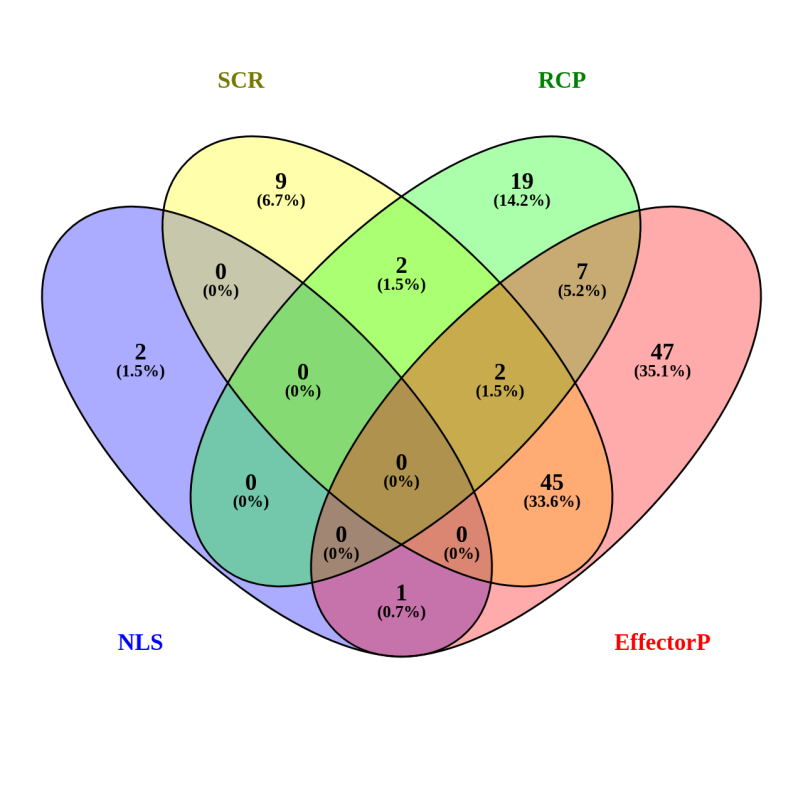
**

**Mp178 Mp4071**

**
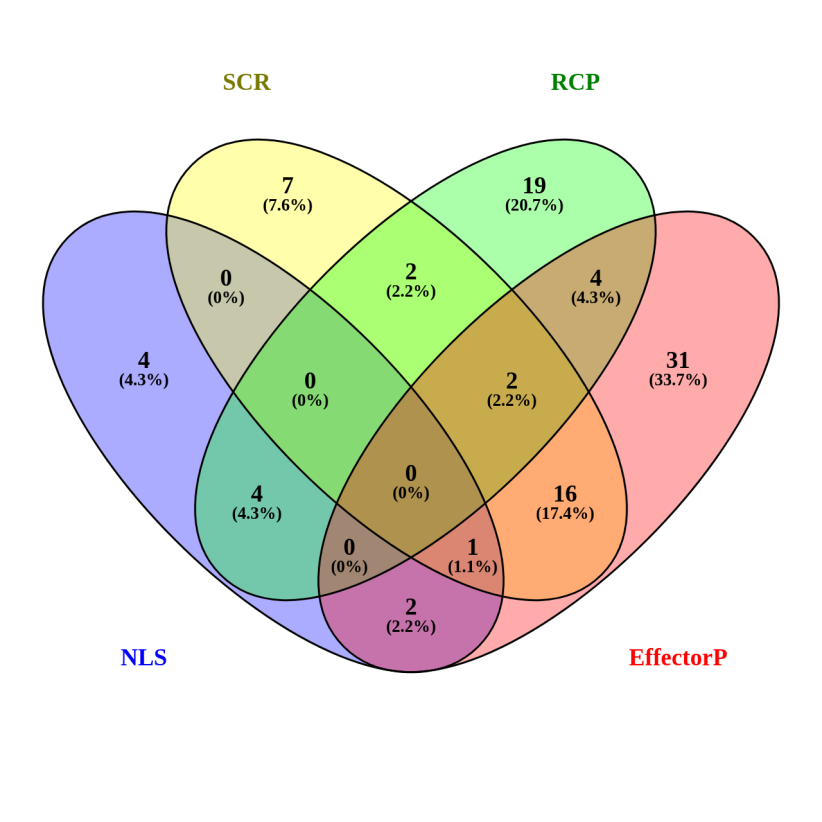

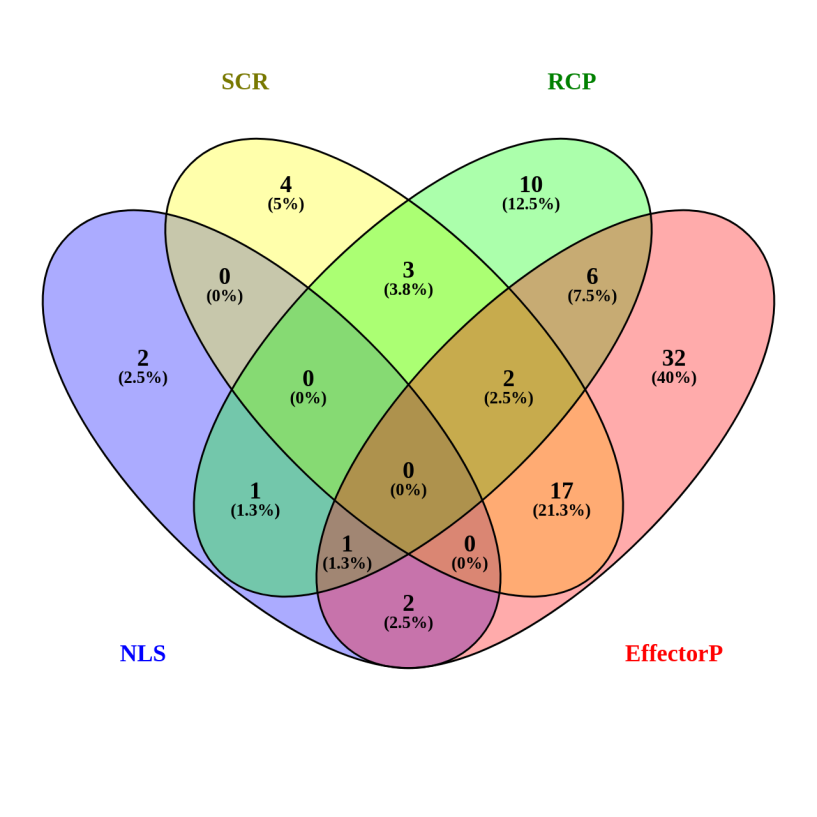
**
